# Bioengineering technologies for enabling biomedical research in space

**DOI:** 10.3389/fbioe.2026.1915463

**Published:** 2026-10-13

**Authors:** Jasmine Gupta, Maedeh Mozneb, Jemima Moses, Arun Sharma

**Affiliations:** 1 Board of Governors Regenerative Medicine Institute, Cedars-Sinai Medical Center, Los Angeles, CA, United States; 2 Department of Biomedical Sciences, Cedars-Sinai Medical Center, Los Angeles, CA, United States; 3 Smidt Heart Institute, Cedars-Sinai Medical Center, Los Angeles, CA, United States; 4 Cancer Institute, Cedars-Sinai Medical Center, Los Angeles, CA, United States; 5 Cexnter for Space Medicine Research, Cedars-Sinai Medical Center, Los Angeles, CA, United States

**Keywords:** 3D bioprinting, bioengineering, biosensors, microgravity, space biology, tissue chips, tissue engineering

## Abstract

Sustained human presence in space requires research platforms capable of capturing the physiological consequences of microgravity, radiation, and isolation, while operating within the severe mass, volume, and crew-time constraints of orbital missions. This review surveys bioengineering strategies that have been adapted to meet these demands: tissue engineering, three-dimensional (3D) bioprinting, tissue chips, and biosensors. Tissue-engineered constructs and human stem cell‐derived organoids provide human-relevant alternatives to animal models and exploit the absence of gravitational settling to generate more uniform, complex multicellular structures, though vascularization and long-term functional stability remain unresolved challenges. 3D bioprinting in microgravity reduces construct collapse and sedimentation-driven defects, and extrusion-based platforms such as the ISS BioFabrication Facility have already demonstrated in-flight tissue printing, though mechanical equivalence to native tissue and photopolymerization-based approaches remain areas of active development. Tissue chips offer a small-footprint, human-specific alternative to traditional cell culture and have been used to model organ systems ranging from cardiac and skeletal muscle tissue to the blood‐brain barrier, supporting both disease modeling and radiation countermeasure testing in orbit. Biosensors, whether deployed independently or integrated with tissue chip platforms, enable real-time, in‐situ data collection that reduces reliance on crew time and sample return. Together, these technologies are converging toward unified, autonomous platforms for tissue fabrication, culture, and monitoring in space. We discuss the current state, limitations, and future directions of each approach, and highlight the opportunities for their integration to advance both astronaut health and terrestrial regenerative medicine.

## Introduction

With the goal to send humans further and for longer in space, the need arises to support the health of future astronauts. The push for commercial spaceflight has increased accessibility to space, making it a viable environment for increased biomedical research and biomanufacturing. The conditions of spaceflight present a unique and harsh environment that can cause significant impact on the human body. Beyond Earth’s protective atmosphere, astronauts encounter microgravity, space radiation, isolation, and confinement which disrupt physiological homeostasis and accelerate cellular and systemic aging (Demontis et al., 2017). While short term missions induce relatively mild effects, extended exposure to these stressors can trigger profound changes throughout the body, including but not limited to musculoskeletal degeneration, cardiovascular deconditioning, and immune dysregulation (Demontis et al., 2017; Sharma et al., 2022; Tomsia et al., 2024). Microgravity alters fluid distribution, leading to extensive adaptations affecting, for example, ocular and cardiovascular function. Optic disc edema, vision changes, and swelling at the back of the eye, also known as Space Associated Neuro-ocular Syndrome have been observed as a result of microgravity (Demontis et al., 2017). With the cardiovascular system, the decreased gravity reduces the amount of work required from the heart which leads to atrophy as well as a reduction in orthostatic tolerance and blood volume throughout the body (The Human Body in Space, 2021). More critically, it disrupts bone and muscle homeostasis, with astronauts losing up to 1% of bone mass monthly due to suppressed osteoblast activity and enhanced osteoclast-mediated resorption (Juhl et al., 2021; Wolfe and Rummel, 1992). Concurrently, skeletal muscle atrophy occurs as microgravity impairs myogenesis and upregulates proteolytic pathways (Juhl et al., 2021; Tomsia et al., 2024). These musculoskeletal changes mirror the effects of terrestrial aging but manifest over weeks to months, offering accelerated insights into degenerative disorders (Demontis et al., 2017; Juhl et al., 2021).

As we venture into deep space exploration away from Earth’s magnetic field and solid shielding, galactic cosmic rays (GCR) and solar proton events could lead to substantial risk of carcinogenesis and degenerative diseases. There is not a comparable alternative on Earth for the high-energy heavy (HZE) ions and the high-linear energy transfer (LET) that the astronauts will face during Moon and Mars missions. Radiation exposure in space further increases risks, damaging DNA and elevating cancer susceptibility, while isolation and confinement impair neurobehavioral health (Tomsia et al., 2024; The Human Body in Space, 2021; Cucinotta and Durante, 2006). Beyond the effects on astronauts’ bodies themselves, microbial behavior shifts in space, with bacteria exhibiting reduced susceptibility to antibiotics, complicating infection management. Collectively, these effects reflect the duality of space as a frontier for exploration and a catalyst for accelerated pathophysiology (Tomsia et al., 2024). Understanding the mechanisms for these adaptations is critical for safeguarding astronaut health and advancing terrestrial medicine, particularly for aging and disuse related conditions (Demontis et al., 2017; Tomsia et al., 2024).

The pursuit of cellular research in space represents an opportunity to address fundamental questions about biological responses to microgravity, radiation, and other extraterrestrial conditions. However, the technical limitations of current methodologies in space—including high costs, operational complexity, and restricted scalability—have historically constrained the accessibility and throughput of such experiments (Van Puyvelde et al., 2025). Terrestrially, bioengineered technologies have developed to expand the range and complexity of devices, tools, and techniques designed to support or improve biological research capabilities. Several of these developments, either already address some of the challenges of conducting research in space or benefit from a microgravity environment. By applying these enabling technologies that enhance cell culture processes in space, we can increase access to orbital research platforms, reduce reliance on resource-intensive protocols, and accelerate the generation of high-quality data. Improved platforms for conducting cell and tissue-based research expand both the capabilities for performing said research as well as the scope of possible experimentation (Ferranti et al., 2020; Van Puyvelde et al., 2025). Higher throughput and greater physiologically relevant data not only promise to enhance our understanding of space-induced cellular changes—such as accelerated aging, altered gene expression, and disease progression—but also pave the way for rapid discovery of countermeasures against space-related health risks. Improved accessibility to efficient, cost-effective cellular experimentation will empower researchers to systematically investigate the mechanisms underlying aging-related pathologies, test novel therapeutics, and advance translational solutions for both astronaut health and terrestrial medicine, bridging the gap between space exploration and biomedical innovation (Ghani and Zubair, 2024a). Addressing these new needs necessitates the development of better and more complex technologies that expand both biological and mechanical capabilities. Currently, bioengineering efforts to meet these needs have focused primarily on tissue engineering, 3D bioprinting, tissue chips, and biosensors.

## Tissue engineering

Tissue Engineering is an interdisciplinary field that combines principles from cell biology, materials science, and biomedical engineering with the goal to develop functional biological engineered tissues that replicate, restore, or improve tissue function (Saltzman, 1997). Tissue engineering can refer to a wide variety of methods to develop specific types of tissues in geometry that either aids or replaces a damaged disease area. As shown in Figure 1, these methods can range from 3-D printed scaffolds or tissues, cultured organoids, organ-on-chip systems, cell-seeded scaffolds, and larger biofabrication systems.

**FIGURE 1:**
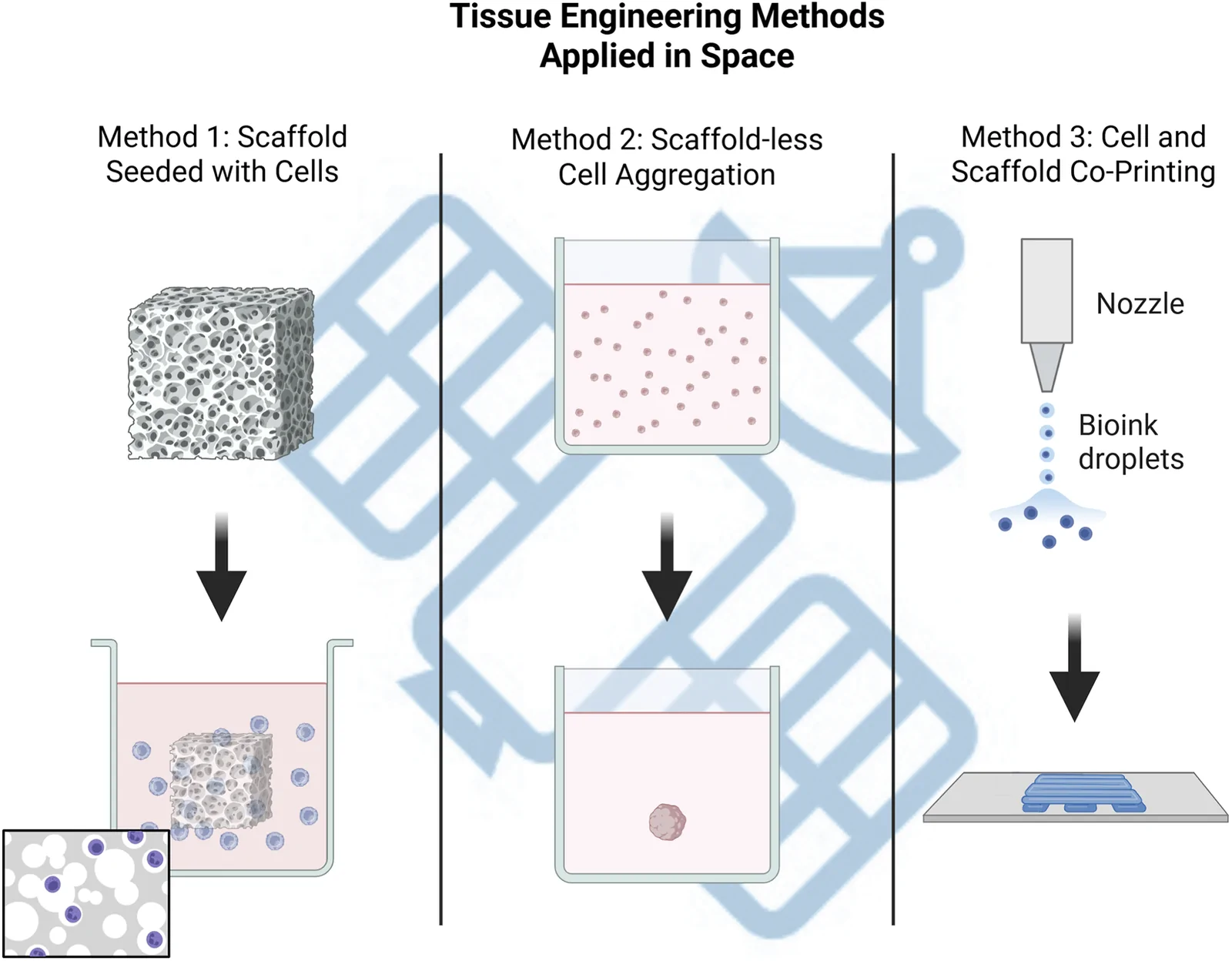
Three major tissue engineering methods: Method one is a 3D porous scaffold that is then cultured within a bath with cells allowing them to seed throughout the scaffold. Method 2 shows the aggregation of cells into a spheroid without the use of a scaffold. Method 3 showcases a 3D bioprinting method that uses a cell-support material jointly extruded into the desired geometry.

Research on tissue engineering in space both benefits from the unique physical conditions of microgravity and simultaneously provides indispensable tools for studying, modeling, and mitigating the physiological challenges of spaceflight. As mentioned, the cost, limited launch opportunities, and practical challenges of conducting human and animal research in space make bioengineered microphysiological systems (MPS) an attractive alternative (Ren et al., 2023). Tissue-engineered constructs serve as human-relevant *in vitro* models for studying the effects of microgravity and cosmic radiation on specific tissues and organs (Ren et al., 2023). Animal models fail to replicate the molecular changes that occur in astronauts, making engineered human tissues and organ-on-chip technology valuable tools (Tavakol et al., 2023). Animal models, while valuable, are expensive both in space and on Earth, fail to recapitulate human physiology due to the absence of human cells and immune responses (Wehland et al., 2025). Human organoids offer a path toward replacing 2D cultures and reducing, if not replacing, animal-based research. As Moroni et al. note, organoids are essentially small “avatars” of a person, providing an opportunity to study authentic human tissue without causing inconvenience or harm (Moroni et al., 2022). Organoids created from human stem cells open up unique possibilities for studying the effects of weightlessness on the function and morphological characteristics of human tissues, the impact of cosmic radiation on cell and organs, and the regenerative potential for human tissues in space (Moroni et al., 2022).

Beyond organoids, specifically engineered tissue models to mimic physiological function have been developed. In order to form the cells into a specific geometry, these engineered models typically include some manner of support either in the form of a scaffold or supportive material in which the cells reside. With the use of an external scaffold, the process either includes the decellularization of an existing tissue or the development of a biocompatible scaffold which is then used as a framework to host and cultivate cells. These seeded cells are then further cultured and allowed to proliferate to the desired geometry, functionality, and maturity of their human tissue counterparts (Jeyaraman et al., 2024).

The study of tissue engineered cartilage has also been significantly studied in space. In a landmark study, Freed et al. cultivated bovine articular chondrocytes on biodegradable polyglycolic acid (PGA) scaffolds in rotating bioreactors. The synthetic scaffolds were manufactured through an extrusion-based method to orient microfibers to form a structure with 97% porosity (Freed et al., 1997; Freed et al., 1994). These scaffolds were seeded with chondrocytes harvested from bovine calves then transferred to the bioreactors. They were first grown for 3 months on Earth, then transferred to the Mir Space Station or maintained on Earth for an additional 4 months. Both environments yielded cartilaginous constructs with viable, differentiated cells that synthesized proteoglycan and type II collagen, demonstrating the feasibility of long-term tissue engineering in space. However, significant differences emerged. Space-grown constructs were more spherical, contained less glycosaminoglycan (GAG), and were mechanically inferior. The spherical shape was attributed to uniform shear and mass transfer at all surfaces in free-floating microgravity, whereas on Earth, gravitational settling of discoid constructs aligned their flat surfaces perpendicular to the direction of motion, increasing circumferential shear and promoting preferential radial growth (Freed et al., 1997). These data are consistent with the idea that tissue function depends on structure (i.e., GAG content), which results from cell activity influenced by mechanical forces that are absent in microgravity (Saltzman, 1997). Subsequent research has confirmed and extended these findings showing these conditions facilitate superior 3D extracellular matrix synthesis and more uniform tissue architecture compared to Earth-based methods. Primary chondrocytes cultured in RWV bioreactors display significant regulation of multiple cartilage and stress-related genes, generating dense, scaffold-free 3D cartilage-like tissues (Jeyaraman et al., 2024). Additional constructs formed from commercial collagen porous sponge scaffolds and donor-obtained human bone marrow mesenchymal stem cells were used to study sex-dependent responses to parabolic flight microgravity (Aissiou et al., 2023).

Tavakol et al. encapsulated induced pluripotent stem cell (iPSC)-derived cardiomyocytes as well as primary cardiac fibroblasts in a fibrin hydrogel which was stretched between two pillars for conditioning. These tissues were electromechanically conditioned for 4 weeks after which they showed cell alignment and striations, contraction in response to adrenergic stimulation, and increased force of contraction in response to increased frequency of stimulation, all pointing to maturation of the tissue (Tavakol et al., 2023). This showcases how support materials, like hydrogels, can be used to support cells in forming a tissue structure. Alternatively, bone marrow tissues were engineered by first seeding decellularized bone matrix with iPSC-derived mesenchymal stromal cells and allowing them to differentiate into osteoblasts over the course of 4 weeks. Human umbilical vein endothelial cells in a fibrin hydrogel and cord blood derived CD34^+^ hematopoietic cells (HSPCs) were then added to the bone matrix. These tissues were evaluated for morphological features and stromal/hematopoietic markers. After 2 weeks of culture, the maintenance of HSPCs and differentiation of myeloid subpopulations were validated, showing that decellularized scaffolds serve as a functional structure within which to engineer tissue. These tissues, validated to represent the desired structures, cell populations, and behavior, were then used to study the effects of cosmic radiation (Tavakol et al., 2023).

The physical environment of microgravity alters the forces acting on cells and developing tissues. Eliminating such a fundamental force as gravity facilitates the formation of complex, large-scale multicellular organoids and enhances specific differentiation pathways (Ren et al., 2023). The absence of sedimentation and buoyancy-driven convection promotes uniform cell distribution through developing constructs which particularly improves difficult to engineer tissues (Sepahvandi et al., 2025). Recently, a commercial company, Lambdavision, has been working to engineer an artificial retina in space. Constructed from layers of bacteriorhodopsin, they are using microgravity to reduce manufacturing inconsistencies caused by gravity (Smith, 2026). A specialized payload has been developed to flow homogenous layers of alternating solutions over the scaffold to build up the artificial retina. On Earth, sedimentation and turbulent flow due to convection cause uneven layers that need to be discarded. Manufacturing space reduces those defects and therefore minimizes waste (Smith, 2026).

Three-dimensional tissue models generated under microgravity conditions also offer platforms for drug screening with significantly greater physiological relevance than conventional 2D cultures (Barzegari and Saei, 2012). Cancer research has been a particular focus of this application, with 3D models of various cancers cultured in microgravity enabling the study of their development, biology, and metastasis in environments that better mimic the *in vivo* cancer phenotype. These models are suitable for high-throughput screening of anticancer drug candidates, and heterogeneous 3D assemblies of cancer and immune cells provide platforms for testing immunotherapeutic strategies (Barzegari and Saei, 2012). Wehland et al. reported that patient-derived colorectal cancer organoids cultured under simulated microgravity exhibited altered morphology, increased viability, and enhanced sensitivity to 5-fluorouracil (5-FU), demonstrating the therapeutic implications of microgravity-conditioned models. Microgravity-generated spheroid models in cancer research offer valuable insights into metastasis, ECM formation, and alterations in gene and protein expression patterns crucial for drug development (Wehland et al., 2025).

One limitation across all tissue engineering is generating functional vascular networks within engineered constructs. Without adequate vascularization, thick tissues cannot survive post-implantation, and patient-response variability remains an unresolved concern. While microgravity may enable more fluidic channels during biofabrication, translating structural advantages into functional, perfusable vascular networks in implantable tissues remains an area of active investigation (Moroni et al., 2022). Additionally, results of engineered muscle tissue and the Mir cartilage experiment show that microgravity induces both positive and negative effects specific to each cell type. Additionally, a major challenge lies in ensuring the long-term viability and functionality of tissues engineered in microgravity once they are reintroduced to Earth’s gravity (Jeyaraman et al., 2024). Longitudinal molecular profiling of engineered tissues, through transcriptomics, proteomics, and metabolomics measures, could complement structural and functional measurements by distinguishing transient adaptation from progressive changes associated with prolonged microgravity exposure. Finally, with multiple methods of simulated microgravity, varying and sometimes conflicting results raise the question of methodological standardization. Ground-based systems produce distinct mechanical and hydrodynamic environments that vary from machine to machine and may even depend on position within each machine (Sepahvandi et al., 2025). Rotary cell culture systems and rotating wall vessels maintain scaffold and cells in a state of constant freefall, effectively reducing sedimentation and replicating key aspects of microgravity. This unique environment supports enhanced cell-scaffold interactions, improved mass transport, and mechanical stimulation, all crucial factors to tissue development (Zenobi et al., 2025). However, one factor in simulated microgravity environments that is not necessarily experienced in true microgravity is mechanical stimulation. Parameters including rotation speed, size of microbeads and organoids, and vessel aspect ratio must be carefully optimized to emphasize the effects of simulated microgravity while minimizing complex mechanical loads (Ren et al., 2023). Dynamic conditions in rotating bioreactors produce non-uniform flow regimes that may affect cell response locally, with cells at the center versus the periphery of the vessel experiencing different shear stress and nutrient transfer (Zenobi et al., 2025). This culture environment also contributes to mechanotransduction, a process by which cells sense and respond to mechanical stimulus, influencing proliferation, morphology, gene expression, and matrix production (Zenobi et al., 2025). All of which could affect the resulting outcome of the experiment and confound the effects of true microgravity on engineered tissues.

## 3D bioprinting

3D printing encompasses a range of methods to either deposit or crosslink a material to form a scaffold or cell-scaffold composite for the purpose of constructing tissue in a specific geometry. It typically uses a layer-by-layer deposition method that places living cells, biomaterials, and biomolecules into a 3D tissue-like structure. The methods for doing so vary but include extrusion, inkjet, laser-assisted, DLP/SLA, microfluidic-based systems, embedded bioprinting such as FRESH and SWIFT, and volumetric bioprinting. There are two main arms of bioprinting: one where cells in a matrix are directly extruded into the desired shape and second where a scaffold with the desired microarchitecture is fabricated then seeded with cells (Agarwal et al., 2025; Zenobi et al., 2025).

All of these varieties of fabrication methods come with specific benefits as well as result in their own unique challenges, especially when used in microgravity, making the choice between them highly dependent on the desired use case. 3D bioprinting in microgravity is an especially promising method for tissue engineering because the conditions of microgravity address a couple of the major challenges with bioprinting on earth. Microgravity reduces cell sedimentation and the gravity-driven collapse of constructs, making it a better environment to print soft or complex shaped constructs (Agarwal et al., 2025; Klarmann et al., 2024). The lower rate of sedimentation results in a more even printing of scaffolds or structures with cells integrated in the material, as well as eliminates the time sensitivity of longer or larger constructs. On earth, the material that is printed is constrained not only by the size of the print, but also by the yield strength of the material to be able to withstand the force of the layers above it. Printing in microgravity results in structures and materials that are able to withstand a lower viscosity (Van Ombergen et al., 2023).

One of the most prominent 3D printing methods is extrusion-based printing. These include direct writing, coaxial extrusion, and printing in a bath which allow for either a scaffold material or scaffold and cell mixture to be placed in a specific geometry for each layer. Direct writing and co-axial extrusion involve the deposition of a material on a print bed whereas printing in a bath involves extruding a material directly into a well full of liquid. That liquid can either simply support the extruded structure or induce solidification of the extruded material (Van Ombergen et al., 2023). Even within this type of printing, there exists a broad spectrum of methods and capabilities, depending on the specific material that is used and the intended outcome for the print. Terrestrially, extrusion based bioprinting is primarily challenged by potential cell damage and low structural integrity of the constructs. A careful balance is needed to develop a bioink that both can maintain its structure and the cell density within it as well as reduce the pressure the cells face during extrusion and risk of nozzle clogs.

Direct extrusion of a support material-cell solution was initially demonstrated with office inkjet printers and later demonstrated on the International Space Station (ISS) using an Epson 800 printer to create precise, thin tissue structures (Van Ombergen et al., 2023). This bioink jet printing benefits from microgravity because a lower viscosity of bioink can be extruded, inducing less shear stress on the cells and a better viability of the overall structure after the print. This lower viscosity, as well as the combined lack of sedimentation, results in less clogging of nozzle when printing (Tabury et al., 2023). Bioinks, which contain both a scaffolding support material as well as cells, require shear-thinning behavior, which is easier to achieve in the microgravity environment. There also is the option of further cross-linking the structure after extrusion, leading to additional stability and long-term viability (Van Ombergen et al., 2023).

The benefit of getting inkjet bioprinting to work includes the customizability of these materials to account for different cell types and material properties of different layers, increasing the range of biological structures that can be printed. Additionally, since each cell type is being directly extruded, the amount of bioink and cells needed to successfully print is minimized to the volume of the print. However, it also raises a few additional challenges in microgravity. For example, during preparation of the bioink, the initial suspension of cells can cause bubbles to appear that will not dissipate or collect in microgravity, potentially resulting in gaps in the construct. Additionally, any material that is extruded and does not attach to the printed structure may float away, attach elsewhere, or simply remain in place blocking later ink, resulting in an inaccurate or failed print (Mo et al., 2024; Van Ombergen et al., 2023).

Currently, the International Space Station houses the ISS BioFabrication Facility (BFF) developed by Redwire Corporation, which has been used to test the printing of several tissue types (Chang and Boland, 2022; Fang et al., 2025). Cardiac tissue has been tried with several projects but has been found to be particularly challenging (Tabury et al., 2023; Van Ombergen et al., 2023). Meniscus tissue, on the other hand, was successfully printed on the ISS. Though the BFF is an extrusion-based printer, Klarmann et al. found that when trying to deposit the collagen and chondroitin-based hydrogel, the ink tended to run up the sides of the needle rather than sit on the print bed. Therefore, they utilized the multi-ink properties of the BFF and 3D printed a well structure and dispensed an agarose support bath into the well. The printer then switched to the bioink and proceeded to print the meniscus tissue (Klarmann et al., 2024). These 3D printed tissues displayed good overall shape fidelity, dimensions comparable to ground controls, and good cell distribution, showing proof of concept for 3D bioprinting in space. The main weakness of the print was the mechanical performance: the meniscus showed a much lower Young’s modulus than the ground control, about 9.8 kPa versus 38.7 kPa (Klarmann et al., 2024). So, structurally and biologically, the print was a success but not yet mechanically equivalent to the ground control.

Zenobi et al. demonstrated that polylactic acid (PLA) scaffolds replicating physiological and osteoporotic-like trabecular architectures could be fabricated with precise control over porosity, interconnectivity, and mechanical properties (Zenobi et al., 2025). The scaffold was fabricated in a standard fused deposition modeling 3D printer using commercial PLA filament. This scaffold was then sterilized and seeded with cells in a rotary cell culture system bioreactor. Successful seeding required the rotational speed of the bioreactor to keep the scaffolds in suspension, a condition that naturally occurs in microgravity. The osteoporotic-like scaffolds, characterized by approximately 80.53% porosity compared to approximately 44.7% for physiological scaffolds, supported osteoblast-like cell adhesion and growth, with an approximately threefold increase in colonization observed under dynamic simulated microgravity conditions. The absence of detectable inflammatory responses, confirmed through tumor necrosis factor-alpha secretion assays, validated the biocompatibility of the integrated scaffold-bioreactor system (Zenobi et al., 2025).

Outside of extrusion-based bioprinting, there are other methods like digital light processing (DLP) and stereolithography (SLA) that solidify the required geometry by inducing cross linking at specific locations and shapes to form that layer of the print. This can be done using lasers or UV. DLP and SLA bioprinting retains much of the same benefits regarding cell settlement within the bioinks as extrusion based bioprinting. One challenge with these volumetric-based printing methods is their requirement for a full bath of the material which is only solidified in specific locations. These 3D printing methods are attractive options for bioprinting because the lack of nozzle reduces the risk of damage for fragile cells (Agarwal et al., 2025; Van Ombergen et al., 2023). In addition, they are much faster and have higher resolution, increasing the potential for high-throughput manufacturing (Agarwal et al., 2025). However, these methods are limited by the optical control of the device and constrained in the materials that can be used. DLP and SLA require photopolymerizable bioinks, have limits on cell loading, and due to the nature of the method, are limited in multi-material capabilities (Agarwal et al., 2025; Van Ombergen et al., 2023). In space, the large bath can cause challenges due to difficulty with containment for the liquid as well as general liquid handling. It also requires much more space and necessitates the use of a singular material or cell type in the print unless a change of material in the bath is completed. Perhaps more challenging than the amount of resources needed to fill the bath during printing, is the requirement for that entire volume to sustain live cells. This further increases the mass required to operate the printer and thus reduces the scalability of such projects in space. Though limited testing has occurred on the ISS, these methods have mostly focused on simulated microgravity experiments due to the challenges and resources required to send a large liquid volume-based 3D printer to the International Space Station.

Using magnetic levitation as the method of simulated microgravity, a glioblastoma model formed from cancer and stem cells was printed using a laser beam assisted bioprinter. This method used a laser beam to solidify the desired shape within a bath, which required an excess of materials and generated significant waste, as well as inducing more stress on the structure due to post cleaning protocols (Rezapour et al., 2023). Additionally, the magnetic levitation method uses gadolinium, which is toxic to cells, adding an extra layer of challenge when simulating microgravity with this method (Rezapour et al., 2023). On the positive side, the laser-based printing method is the least affected in microgravity since laser function does not change in the space environment, meaning that less customization is needed to get it to work in space. With magnetic levitation in microgravity, no scaffold is needed, giving precise control over the deposition and placement of cells (Sarabi et al., 2022).

Overall, there are numerous methods of 3D bioprinting that have been shown to successfully produce tissue structures. Extrusion-based printing is well developed and validated, making it a strong option for space-based bioprinting. The use of a support bath, while potentially introducing some challenges in the containment, has been shown to mitigate some of the detrimental fluid handling effects of microgravity, resulting in a smoother print. Photopolymeric printing methods show promise for high-throughput manufacturability and precise architectures but are limited in material and biological complexities. Future expansion of space bioprinting capabilities relies on technological and methodological improvements to reduce material mass, waste, and errors that would require manual intervention (Van Ombergen et al., 2023). The 3D printing device would require a fully enclosed chamber to contain the multiple liquids used in the process as well as sensors for temperature, oxygen, and pH levels to maintain the tissue during the print and parallel culture chambers for post-print maturation of the tissues (Shah et al., 2025; Van Ombergen et al., 2023).

## Tissue chips

Tissue chips are microfluidic devices that enable precise control over fluid movement and enable unique geometries to culture cells in a tissue-like structure. These microphysiological systems can then be used to study these tissues in a variety of conditions and provide a more physiologically relevant model to analyze (Kong et al., 2026; Mu et al., 2022; Strods et al., 2024). Compared to the monolayer cultures of 2D cell culture, these tissue chips allow researchers to obtain more accurate results from the *in vitro* culture. These devices are especially useful for space biology research in that they require minimal space and resources, are capable of automation, and have the aforementioned physiological relevance (Mu et al., 2022). Their fully contained system also means that they are safer to use in space than traditional culture methods that leave fluids open to air. In addition to supporting general space biology research capabilities, these tissue chips used in conjunction with iPSCs could serve as a personalized platform for understanding the potential effects of space conditions on astronauts and optimizing protective measures (Tavakol et al., 2023).

As such a promising method to fly biological experimentation on the International Space Station, NCATS and the ISS National Laboratory launched the Tissue Chips in Space Initiative galvanizing the push to use tissue chips to study the microgravity environment for disease modeling and to understand the effects of space on the body (Jogdand et al., 2024; Low and Giulianotti, 2020; Yau et al., 2023a). These models have included the brain, gut, heart, liver, neurovasculature, lungs, bone marrow, bone, cartilage, kidneys, and the blood-brain barrier (Kong et al., 2026; Low and Giulianotti, 2020; Mu et al., 2022). Microfluidic chips are incredibly versatile in their design and can be constructed to fit the culturing and post-processing needs of the project. Methods of manufacturing include soft lithography, 3D printing, and injection molding depending on the structure and complexity of the chip design. Currently, tissue chips maintain a fairly simple structure including a few channels in parallel or separated by a porous layer to allow for co-culture. Much of the set up for tissue chip experiments are similar to Earth-based experiments, though extra consideration for automation and effective bubble traps is needed. Figure 2 shows a cross section of one such co-culture chip with a top and bottom channel each with a different cell type separated by a porous section to allow for media and biomolecules to flow between the two channels. These chips are used to study cell-cell interactions or co-cultured responses to external stimuli.

**FIGURE 2:**
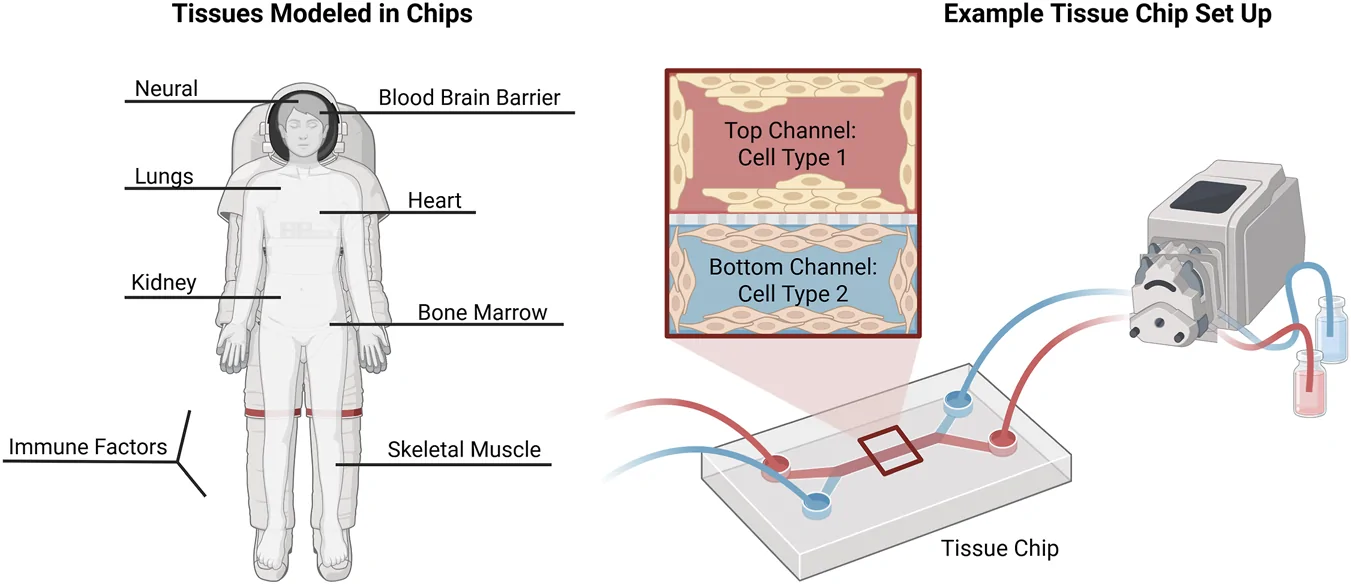
Left: Tissue chips have been used to model all of the identified organs/tissues. Right: An example tissue chip set up with a top and bottom channel and porous membrane separating the two. Either channel can contain different cell types while the pores allow signaling and biomolecule exchange across the membrane. The entire fluidic system is fully contained and can be automated making it ideal for space applications.

Tissue chips modeling skeletal muscle tissue have been flown on multiple payloads, elucidating the downregulation of myoblast proliferation and muscle differentiation. They also showed a decrease in muscle-metabolism pathways and reduced inflammatory signaling. Genomic analysis shows that there are shifts in fiber type and muscle differentiation, reinforcing space as a model for sarcopenia-like phenotypes (Parafati et al., 2023). Cardiac tissue chips have also been launched to study engineered heart tissue and heart tissue arrays to show an array of cardiac conditions like ischemic cardiomyopathy. These models allow for research into understanding these changes in order to potentially prevent or reduce these effects (Low and Giulianotti, 2020). Tavakol et al. developed an organ-on-chip model to understand the effects of high-linear energy transfer (LET) neutron radiation on human tissues, representing the first use of engineered human tissues for this purpose. The study exposed engineered cardiac tissues (eCT) and engineered bone marrow (eBM) to simulated cosmic radiation at doses of 1–6 Gy. Results revealed tissue-specific damage patterns and allowed for proof-of-concept testing of radioprotective agents, including amifostine pre-treatment of eCTs and granulocyte colony-stimulating factor (G-CSF) post-treatment of eBMs, demonstrating that engineered human tissues can serve as individualized, human-relevant alternatives to animal models for space radiation research (Tavakol et al., 2023).

Beyond disease states, space studies of cardiac tissues show angiogenesis, endothelial spheroid formation, and accelerated differentiation of cardiac progenitors (Jogdand et al., 2024). Without microgravity, changes in tight-junction behavior, cell adhesion, fluid flow, and barrier integrity could pose a significant difference in function. Tissue chips are also used to examine the other major conditions of spaceflight, radiation. To model the blood-brain barrier (BBB), tissue chips are a viable platform for space studies. This model is especially useful for understanding cell-cell interactions particularly brain-vascular signaling and neuropathic drug responses (Jogdand et al., 2024; Yau et al., 2023a). Bone marrow chips showed dose-dependent losses of hematopoietic stem and progenitor populations after gamma irradiation that align with live mouse models. The G-CSF partially restored stem and progenitor cell numbers (Mu et al., 2022; Yau et al., 2023a). This shows that tissue chip models are valuable analogs to research space-radiation injury and countermeasure testing.

Tissue chips as a mechanical device benefit space biology research because of the small footprint, biological relevance, multi-cell type capabilities, and potential for automation (Mu et al., 2022; Yau et al., 2023b). On the other hand, these microfluidic chips also have a number of improvements needed to fully utilize the potential of these devices. Perhaps the greatest of these is the automation and integration with biosensors. In order to take OOCs from a culture platform to a biosensing and experimental system, a reliable method of fluid handling and real-time empirical data collection is necessary (Kong et al., 2026; Mu et al., 2022; Yau et al., 2023b). Future chip platforms could additionally incorporate automated sampling and preservation capabilities, enabling transcriptomic, proteomic, metabolomic, or single-cell analyses at defined experimental timepoints while minimizing crew intervention. The ability to switch between multiple reservoirs to supply various reagents without the introduction of bubbles would widen the scope of experiments that can be run in these chips. Additionally, since these chips can be tailored and manufactured for specific uses, the concern about standardization across studies is brought up. Uniform criteria for quality control and disclosure of protocols make it easier for results across different labs and missions to be compared (Yau et al., 2023b). For use in space, the challenges are increased with the chips needing to withstand the launch and return forces and lack of gravity-driven transport.

## Biosensors

With all biological experimentation, the following analysis is what identifies specific quantifiable measures for the changes wrought by the conditions of space. It is the basis of the science, making higher-throughput and automated processes for gathering this data highly beneficial, especially when the alternatives are delayed processing with sample return or time intensive crew operations. *In-situ* measurements also allow for greater accuracy and flexibility of experiments as one can respond to the immediate conditions of the sample.

Biosensors are used to detect and measure both mechanical and chemical outputs by biological samples during an experiment. Factors like contraction and current, as well as analytes like proteins and metabolites can all be tracked and compared over the course of time and across samples to measure the effects of variable conditions on samples (Ferrari et al., 2020; Yau et al., 2023b). The use of these technologies to automatically collect and quantify is especially important for space research where minimal astronaut interaction and collection at discrete timepoints is needed. These sensors have been developed to function individually as well as integrated into existing testing platforms, such as microfluidic organ on a chip models.

External biosensors, unattached to engineered testing platforms, have been developed both as methods for autonomous assays and to collect key biophysical data from astronauts in real time. Terrestrial sensors have been designed to collect images, temperature, oxygen and pH levels (Ferrari et al., 2020). Since research on Earth is not constrained by as many safety, space, and material restrictions, these sensors have much greater flexibility in how they can be constructed and used. Additionally, data collection in labs is often performed by conducting the assay protocols by hand rather than through an automated process. Actual biophysical sensors are more commonly used when collecting data from patients and as part of a full monitoring system.

In space, wearable biosensors can be used to monitor astronaut health and assess the effect of space on the body (Griffith et al., 2023). Griffith et al. designed an electromagnetic resonant skin patch sensor to monitor microgravity-induced hydrostatic pressure gradient changes. By using electromagnetic waves to better penetrate biological tissue, it detects changes in the resonant frequency to alert for the need to implement SANS countermeasures. Tested with both lower body negative pressure and head down tilt conditions and verified through ultrasound images of the optic nerve sheath, this non-invasive measure accurately detected the cranial volumetric fluid shifts (Griffith et al., 2023).

Alternatively, initial testing of biosensors has shown promise for expanding the research conducted in microgravity. Xu et al. developed a super wettable microchip capable of consistently creating controlled individual droplets for further sensing assays. This plate utilized alternating patterns of superhydrophilic wells with a superhydrophobic nanodendritic silica coating to allow for precise droplet control without extensive complexity (Xu et al., 2017). These sample droplets are then used to complete colorimetric detection of markers such as glucose, calcium, and various proteins (Xu et al., 2017).

In addition, Moroni et al. proposed the concept of human organoids, micro-tissues developed from stem cells that exhibit the histological and functional characteristics in mature organs, as biological “sentinels” with built-in capacity to report chemical cytotoxicity or radiation exposure through easily detectable signals like fluorescent proteins. This approach enables real-time biomonitoring of the effects of microgravity and cosmic radiation on human tissues at the molecular and cellular levels (Moroni et al., 2022). Imaging is one of the key metrics to be collected throughout an experiment. Currently on the ISS, it is one of the main methods of collecting real-time data mid-experiment. It is challenging to bring microscopes to the ISS due to the complexity and sensitivity of such an instrument, so the existing on-board microscope is used, requiring manual setup. So, more compact, modular, and autonomous imaging systems are needed to improve science outcomes. One such design contributing to the expansion of imaging capabilities as a base data collection method is the EuniceScope. This modular, 3D printed brightfield microscope shows functional image quality and adaptability to different configurations for space payloads (Chu and Tsia, 2023).

Automating space research even further, biosensors integrated with technologies such as tissue chips improve and expand the range of possible experimentation. As previously mentioned, organ-on-chip or tissue chip experimenting is a promising platform for biological research in space. The contained fluidic system allows for precise and controlled movement of fluid, making it conducive to sensing assays. More complex or connected microfluidic chips can be developed to perform a number of molecular assays, electromechanical activity, or even single cell analysis (Kanapskyte et al., 2021; Xu et al., 2025; Yau et al., 2023b). Figure 3 shows an example experimental system that integrates tissue chips with biosensors and downstream analysis. The main challenge with integrating these technologies will be developing a reliable and accurate chip-based assay while maintaining the small footprint and limited manual operation that makes tissue chips such a promising option (Yau et al., 2023b). The geometry of the channels also allows for integrated circuits. Various porous scaffold and microelectrode configurations have been tested to monitor and control cardiac, neuronal, and muscle tissue function. Similarly, to better capture data, microelectrode configurations for nanoelectronic meshes to detect movement in the tissues from chips have been developed and tested on cardiac organoids. A basket-shaped mesh was developed to enclose the spheroid and therefore increase signaling (Yau et al., 2023b). This electronic mesh was used to create cyborg organoids that integrate the mesh with cells as they grow to form the organoid structure. With minimal impact on the cells, this mesh can be used to monitor the tissues through differentiation and maturation (Li et al., 2019; Yau et al., 2023b).

**FIGURE 3:**
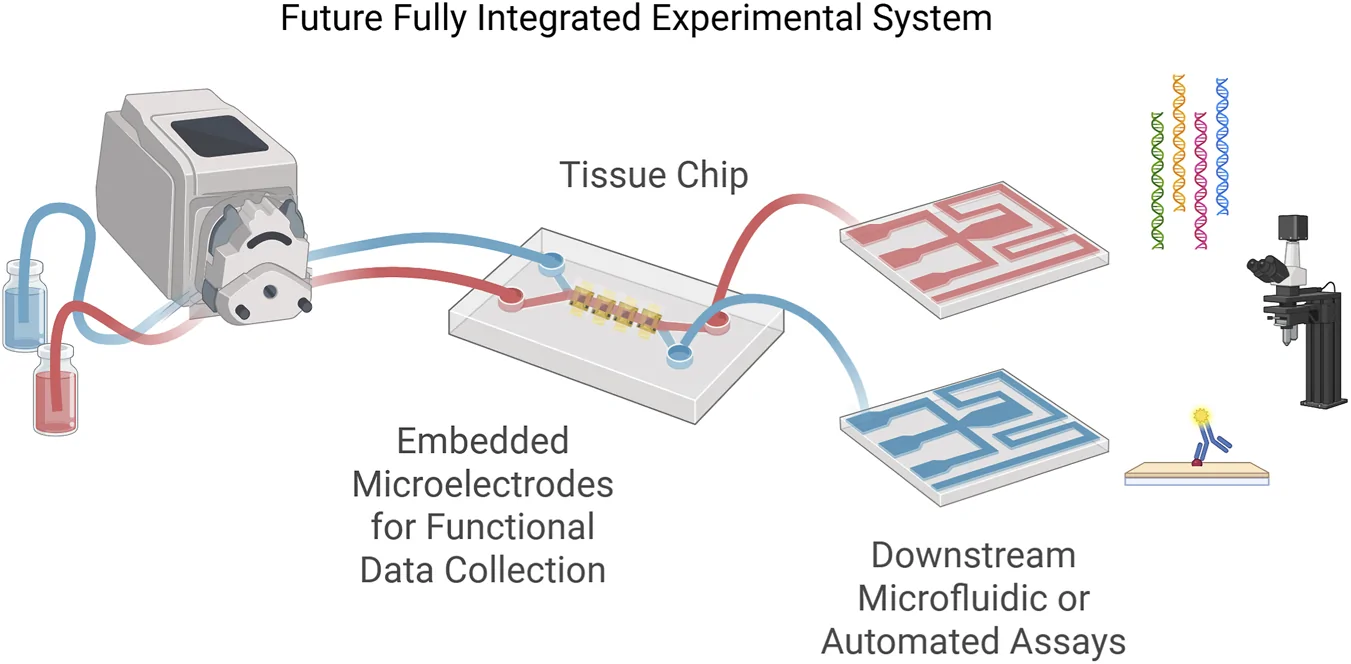
Hypothesized layout for fully integrated experimental system that includes the microfluidic chip seed with cells, embedded microelectrodes to collect real time functional data throughout the experiment, and downstream connected microfluidic or automated assays to analyze the effluent. Sequencing, labeling, and imaging could also be connected downstream.

Though still in the early stages, biosensors hold a large opportunity to expand research capabilities in space. Current research is restricted both in experimental length and complexity with the limitations on data collection mid-experiment. Existing biosensors would need to be adapted to function without gravity-based fluid flow. The development of new and better sensors could exponentially expand the quality and types of data collected. Real-time or even multiple mid-point assessments of cell viability, protein expression, or biomolecule concentration will greatly improve our understanding of the effects of space. The combination of automation and biosensor integration will allow high-throughput studies and minimize crew time, allowing for more experiments to be run. The convergence of all development needs across tissue engineering, 3D bioprinting, tissue chips, and biosensors is summarized in Figure 4, which outlines a roadmap toward integrated space bioengineering platforms and their downstream translational applications.

**FIGURE 4:**
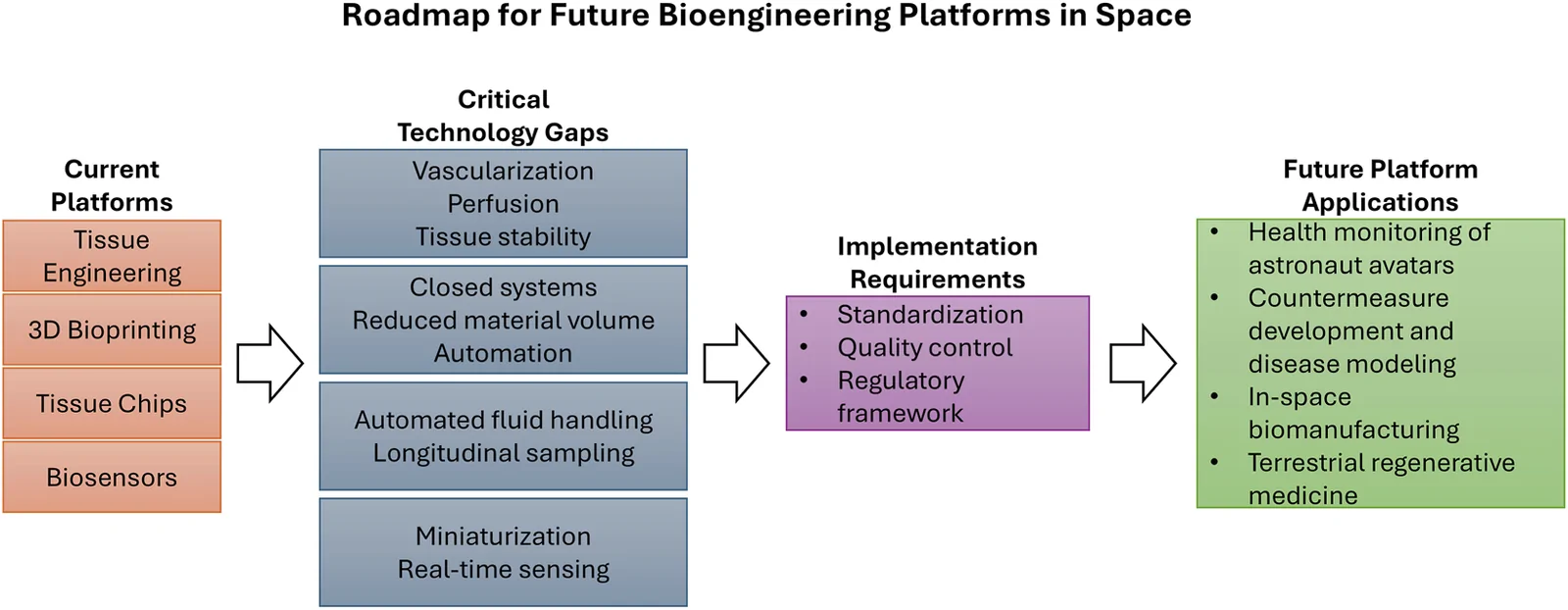
Roadmap for the development of translational bioengineering platforms for space-based biomedical research.

## Regulatory, ethical and legal considerations

Regulatory considerations become critical when it comes to using cell-based therapies on Earth or in-space. On Earth agencies such as US Food and Drug Administration (FDA) ensures safety, purity and consistency of cell- and tissue-based therapies. Under the Outer Space Treaty of 1967 provides a primary constitutional framework for space activities. Jurisdiction and control (Article VIII) state the nation that launches a spacecraft retains jurisdiction and control over that object and any personnel or payloads inside it, meaning terrestrial laws of the launching state apply to the lab bench in orbit (Carranco et al., 2026; Dembling and Arons, 1967). Any form of stem cell production or drug development on the ISS would need to meet the same GMP standards as on Earth with oversight by the responsible national authority.

In the recent years, NASA has engaged with the FDA to brief them on biomanufacturing experiments and align expectations for space-derived therapeutics in the future. In a study conducted by Mayo clinic in 2020, mesenchymal stem cells (MSCs) grown on the ISS were characterized extensively during post-flight analysis to verify the space-grown cells met safety benchmarks (Huang et al., 2020). Stem cells grown in space need to have specific protocols and safety checkpoints unique to space. Expanding stem cell for regenerative medicine is a novel therapeutic strategy, quality control procedures and regulatory guidelines need to be established based on identity, purity, sterility, safety and potency (Ghani and Zubair, 2024b). Based on terrestrial human research ethical frameworks should be built around principles like informed consent, scientific excellence and social responsibility. Additionally international law has yet to catch up, emerging space law alliances and conferences (Space Beach Law, 2026) aim to better protect human subjects, data privacy and intellectual property rights in space. Dialogues between space agencies, bioethicists and policymakers are underway to ensure that we translate space biomedicine research into applications, safely, ethically and with appropriate legal frameworks (Mozneb et al., 2025).

## Conclusion

As human space exploration extends toward longer-duration missions and deeper space environments, the physiological challenges imposed by microgravity, radiation, and isolation demand innovative and scalable research solutions (Iosim et al., 2019). Not only that, but the operational constraints of mass, volume, risk mitigation, and limited crew time require even existing technology on Earth to be reconfigured for space usage (Nangle et al., 2020). Tissue engineering, 3D bioprinting, tissue chips, and biosensors collectively address these challenges by enabling more physiologically relevant, high-throughput, and automated biological investigations in space. Together, these technologies enable human-relevant models that can capture mechanobiological, metabolic, immunological, and radiation-induced responses with far greater fidelity than conventional 2D culture systems.

Each technology offers distinct advantages while also presenting critical limitations. Tissue engineering and organoid systems provide human-relevant models that surpass traditional animal studies. Because microgravity removes gravitational settling and reduces density-driven transport limitations, it often allows for a more uniform cell distribution, enhanced spheroid formation, and improved three-dimensional organization (Freed et al., 1997; Ren et al., 2023; Smith, 2026). However, its broader utility is still limited by incomplete vascularization, inconsistent long-term maturation, and uncertain functional stability after returning to 1g conditions (Jeyaraman et al., 2024; Klarmann et al., 2024).

3D bioprinting is similarly attractive because microgravity reduces construct collapse, sedimentation, and nozzle-related limitations associated with gravity-dependent printing. This expands the design space for soft, delicate, and geometrically complex tissues while also lowering some constraints on bioink viscosity and shape fidelity. Extrusion-based methods have reached sufficient maturity for flight validation, as demonstrated by successful meniscus tissue printing on the ISS BioFabrication Facility, though mechanical equivalence to native tissue has not yet been achieved (Sepahvandi et al., 2025). Photopolymerization-based methods offer higher resolution and throughput potential but remain constrained by material requirements and the logistical challenges of containing large liquid volumes in microgravity (Demontis et al., 2017; Yau et al., 2023b). Still, 3D bioprinters face challenges in mechanical robustness, fluid handling and containment, and material constraints.

Tissue chips offer one of the strongest platforms for space research because they combine a small footprint, controlled perfusion, multicell co-culture, and human-specific biology in a format that is compatible with constrained mission resources. They are especially well suited for studying organ-level phenotypes, pharmacologic responses, and radiation injury under tightly defined conditions though standardization and integration with sensing systems remain ongoing hurdles (Freed et al., 1997; Smith, 2026).

Biosensors, particularly when integrated with these platforms, support *in situ* data acquisition that is essential for reducing crew intervention and enhancing experimental resolution. Real-time monitoring of metabolites, protein expression, electromechanical activity, and cell viability addresses the fundamental limitation of discrete timepoint sampling (Ghani and Zubair, 2024a; Aissiou et al., 2023; Li et al., 2019). Though a start has been made to create these sensors, further development is needed to achieve reliability, miniaturization, and seamless integration with other bioengineered technologies. The breadth of sensors that have not been developed yet in conjunction with the extensive benefits from the integration of biosensors makes this a key opportunity for improvement within bioengineered solutions of space biology research.

Moving forward, the greatest impact will likely arise from the integration of these technologies into unified, autonomous systems capable of supporting experiments from tissue fabrication and culture to monitoring and analysis. Such a platform would allow space researchers to generate more reproducible data, test countermeasures faster, and probe deeper into the effects of space on human biology. Advances in automation, standardization, and scalability will be essential to maximize experimental throughput and reproducibility in the constrained environment of space.

Ultimately, space-based bioengineering research not only supports the future of human spaceflight but also accelerates innovation in biomedical science on Earth. By leveraging the unique environment of space as both a stressor and a tool, these technologies provide the opportunity to study disease mechanisms, test therapeutics, and develop regenerative solutions. As these platforms mature, they will play a central role in bridging the gap between experimental biology and clinical translation, benefiting both astronauts and patients alike.
